# Two case reports of medication-induced elkonyxis in adolescents and literature review

**DOI:** 10.1016/j.jdcr.2026.06.024

**Published:** 2026-06-18

**Authors:** Nesreen Shahrour, Lillian Bleicken, Alexandra Gosh, Robert A. Silverman

**Affiliations:** a Georgetown University School of Medicine, Washington, District of Columbia; b Michigan State University College of Osteopathic Medicine, East Lansing, Michigan; c Department of Dermatology, MedStar Washington Hospital Center/Georgetown University Hospital, Washington, District of Columbia

**Keywords:** adolescent nail dystrophy, drug induced nail disease, elkonyxis, infliximab, isotretinoin, nail matrix injury, nail unit inflammation, onychoatrophy, onychomadesis, retinoid toxicity, TNF inhibitor induced psoriasis

## Introduction

Elkonyxis is an uncommon nail plate defect characterized by a large focal well-circumscribed depression or punched out area that begins at the lunula, and arises most often from injury at the proximal nail fold or nail matrix.^1^ It is a distinctive form of nail plate atrophy. Some medications have been associated with increased nail matrix fragility by altering keratinization or by inducing inflammatory disease in the periungual unit. The literature suggests that chemotherapy agents and oral isotretinoin are associated with nail matrix disruption.^2^ The nail matrix is formed of proliferating cells and chemotherapy agents arrest this proliferation and lead to abnormal keratinization.^2^ Isotretinoin is a derivative of vitamin A used to treat acne vulgaris resistant to conventional treatment. It is associated with various side effects, including nail changes.^3^ Systemic isotretinoin modulates nuclear retinoid receptors in nail matrix keratinocytes, altering keratin expression, increasing keratinocyte turnover, and reducing intercellular cohesion.^3^

Few publications describe elkonyxis in adolescents in a medication-associated context. We describe 2 adolescents with elkonyxis as the dominant nail finding, 1 temporally associated with isotretinoin exposure and nail trauma from acrylic removal, and 1 occurring in the setting of infliximab-associated paradoxical psoriasiform eruption.

## Case 1

A 17 year old female receiving isotretinoin for acne vulgaris developed painful nail changes after removal of acrylic nails. At presentation, the patient received a cumulative isotretinoin dose of 77 mg per kg. Examination showed pyogenic granuloma-like granulation tissue extending from the proximal nail fold of the right thumbnail and elkonyxis of the left thumbnail, with focal proximal plate loss rather than diffuse nail atrophy (Fig 1, *A*). Elkonyxis is a rare side effect while on isotretinoin, with few reports in the literature.^4^ Retinoid-associated periungual granulation tissue and pyogenic granuloma-like lesions have been reported during isotretinoin therapy in younger patients, supporting that the periungual tissue response can be drug associated rather than purely traumatic.^5^

**Fig 1:**
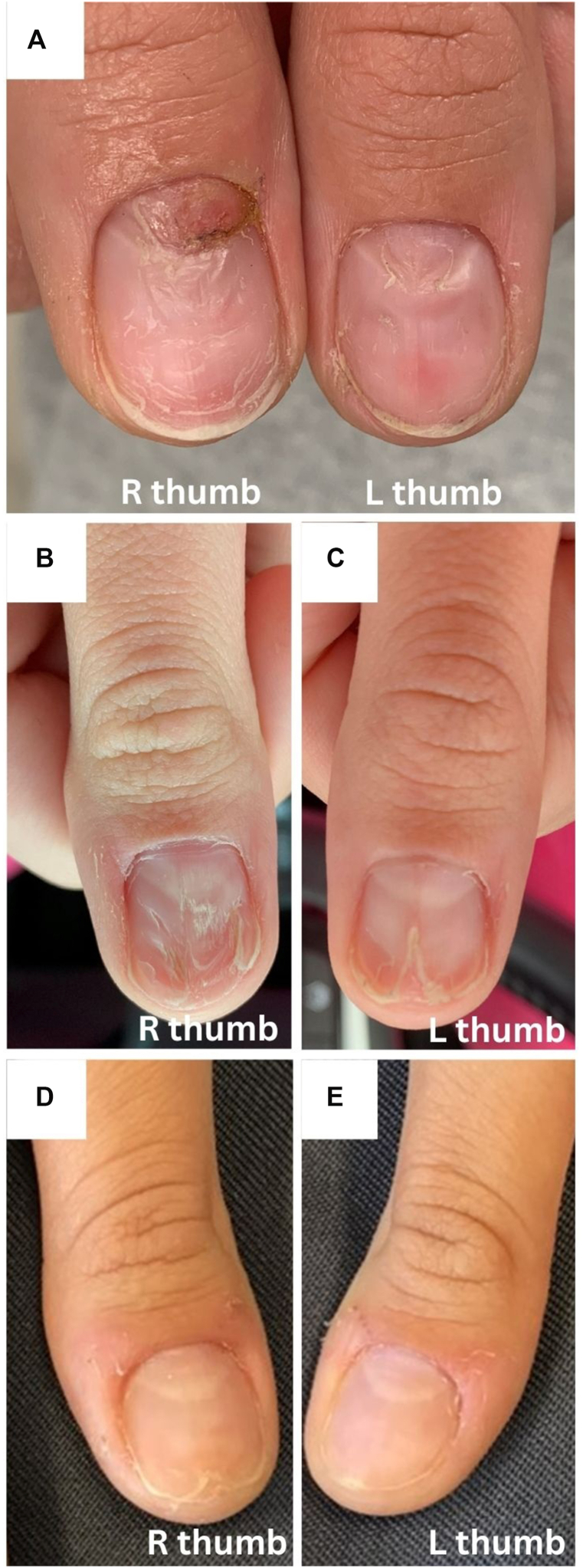
**A,** Pyogenic granuloma-like granulation and elkonyxis: First in-person clinic presentation showing pyogenic granuloma-like granulation of proximal right thumbnail and focal proximal elkonyxis of left thumbnail on right of picture. **B,** Onychorrhexis and atrophy: Right thumbnail presenting with onychorrhexis and nail atrophy 2 months post-treatment initiation. **C,** Median nail dystrophy: Left thumbnail presenting with median nail dystrophy 2 months post-treatment initiation. **D,** Resolution of elkonyxis: Right thumbnail almost completely healed 6 months post-treatment initiation. **E,** Resolution of elkonyxis: Left thumbnail almost completely healed 6 months post-treatment initiation.

Clobetasol 0.05% cream was applied twice daily for 2 weeks. Two months later, nail atrophy and onychorrhexis were present in the right thumbnail, while median nail dystrophy was present in the left thumbnail (Fig 1, *B* and *C*). A second 2-week course of clobetasol 0.05% cream twice daily was recommended. An over-the-counter nail strengthener was suggested for the remainder of the isotretinoin course and for 3 additional months until normalization. In this case, mechanical injury from acrylic removal likely disrupted the proximal nail unit, while isotretinoin related alterations in keratinization enhanced nail matrix fragility.

## Case 2

A 13 year old male with ulcerative colitis on infliximab for more than 1 year developed a widespread psoriasiform eruption and concurrent nail involvement. Well-defined erythematous papules and plaques with micaceous scale were distributed on the scalp, trunk, flexures, and acral surfaces and involved approximately 10% body surface area. Nail examination showed elkonyxis of the left thumbnail, characterized by a focal well-circumscribed proximal plate defect without complete nail plate shedding (Fig 2).

**Fig 2:**
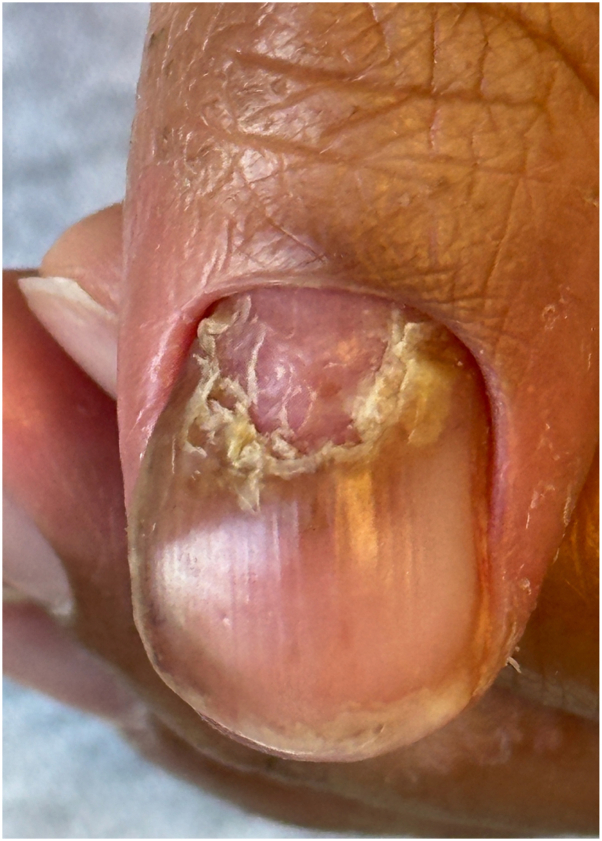
Elkonyxis: Left thumbnail displaying a well-circumscribed proximal plate defect with focal thinning and loss of nail plate over the lunular region, exposing an erythematous nail bed with adherent crust and granulation tissue.

Two weeks before presentation to the dermatology clinic, infliximab was discontinued, and therapy was transitioned to ustekinumab by his gastroenterologist due to concern that his cutaneous manifestations were secondary to infliximab. Testing for infliximab antibodies was negative. Prior topical therapy with high potency corticosteroids and lower potency corticosteroids on sensitive areas provided limited relief. Topical roflumilast 0.3% cream was added to his regimen for use on the left thumbnail and hands, and roflumilast 0.3% foam was used for the scalp. At follow-up 6 months later, the patient’s skin and nail findings improved substantially. Sixteen weeks after changing treatments to ustekinumab and topical roflumilast, elkonyxis had resolved leaving only residual trachyonychia.

## Discussion

Both of these cases illustrate medication-associated nail matrix injury presenting as elkonyxis. In Case 1, isotretinoin exposure temporally preceded the onset of matrix level dystrophy following acrylic nail removal. In Case 2, elkonyxis developed alongside a new psoriasiform eruption while the patient was receiving infliximab and improved after discontinuation of infliximab and transition to ustekinumab, supporting a drug associated inflammatory process. Onychomadesis and onychoatrophy were on the differential. However, the clinical morphology was not dominated by complete proximal nail plate shedding or diffuse nail plate underdevelopment.

The pathophysiology in Case 2 is consistent with tumor necrosis factor (TNF) inhibitor-induced paradoxical psoriasis. Infliximab blocks TNF-alpha, which normally limits plasmacytoid dendritic cell production of type I interferons. Therefore, TNF blockade can cause excess type I interferon signaling, activating dendritic cells and downstream interleukin 23 and T helper 17 pathways.^6^ This cascade drives keratinocyte hyperproliferation and psoriasiform inflammation. Clinically, patients can develop well-demarcated erythematous plaques with scales that are histologically and morphologically similar to psoriasis.^7^

Management of these patients should align with feasibility and disease control. For isotretinoin associated nail matrix fragility without systemic complication, topical corticosteroids and discontinuation of traumatic nail practices can be reasonable while continuing isotretinoin as elkonyxis likely will resolve when the course of isotretinoin is complete.^8^ For TNF-inhibitor induced paradoxical psoriasis, evidence supports skin-directed therapy when disease is mild, then consider switching within class or switching out of class for refractory, severe, or quality of life limiting disease.^8^ Elkonyxis is differentiated from nail pitting seen in psoriasis, as pitting consists of small, shallow depressions, whereas elkonyxis refers to larger, deeper punched out defects. A review found 127 patients receiving TNF inhibitors with induction or exacerbation of psoriasis. Topical corticosteroids resolved 26.8% of cases. Switching TNF agents resolved 15.4% and stopping TNF therapy resolved 64.3% of cases.^9^ Additionally, a case series of 7 patients with nail psoriasis treated with roflumilast 0.3% cream reported clinical improvement, which may have contributed to improvement in our patient.^10^

These cases demonstrate that elkonyxis can be a clinical marker of medication-associated nail matrix injury in adolescents exposed to systemic agents that disrupt either keratinocyte differentiation or immune homeostasis. Our observations highlight 2 distinct but convergent pathways to nail matrix dysfunction and support that the presence of elkonyxis should prompt medication review, assessment for mechanical or cosmetic trauma, and early management, as recognition of this focal nail finding may allow timely counseling and therapeutic adjustment to prevent progression of drug-induced nail disease.

## Conflicts of interest

None disclosed.
